# Use of Sodium Bicarbonate During Pediatric Cardiac Admissions with Cardiac Arrest: Who Gets It and What Does It Do?

**DOI:** 10.3390/children6120136

**Published:** 2019-12-16

**Authors:** Rohit S. Loomba, Mubbasheer Ahmed, Mubeena Abdulkarim, Enrique G. Villarreal, Saul Flores

**Affiliations:** 1 Division of Cardiology, Advocate Children’s Hospital, Oak Lawn, IL 60453, USA; mubeena.abdulkarim@advocatehealth.com; 2 Department of pediatrics, Chicago Medical School, Chicago, IL 60612, USA; 3 Critical Care and Cardiology, Pediatrics, Texas Children’s Hospital, Houston, TX 77030, USA; mahmed@bcm.edu (M.A.); enrique.villareal@bcm.edu (E.G.V.); saul.flores2@bcm.edu (S.F.); 4 Department of pediatrics, Baylor College of Medicine, Houston, TX 77030, USA; 5 Tecnologico de Monterrey, Escuela de Medicina y Ciencias de la Salud, 64849 Monterrey, Nuevo Leon, Mexico

**Keywords:** sodium bicarbonate, pediatrics, heart arrest, pediatric intensive care units, congenital heart disease, cardiopulmonary resuscitation, resuscitation, mortality

## Abstract

The objectives of this study were to characterize the use of sodium bicarbonate in pediatric cardiac admissions that experience cardiac arrest, to determine sodium bicarbonate use over the years, and to determine the impact of sodium bicarbonate on length of admissions, billed charges, and inpatient mortality. A cross-sectional study was conducted utilizing the Pediatric Health Information System database. Characteristics of admissions with and without sodium bicarbonate were initially compared by univariate analyses. The frequency by which sodium bicarbonate was used was compared by year. Regression analyses were conducted to determine the impact of sodium bicarbonate on length of stay, billed charges, and inpatient mortality. A total of 3987 (50.3%) of pediatric cardiac intensive care admissions with cardiac arrest utilized sodium bicarbonate; however, frequency changed from 62.1% in 2004 to 43.7% in 2015. Linear regression analysis demonstrated a decrease in length of stay (−27.5 days, *p* < 0.01) and billed charges (−$470,906, *p* < 0.01). Logistic regression analysis demonstrated an increase in mortality (odds ratio 1.77, 95% confidence interval 1.56–2.01). In conclusion, sodium bicarbonate is being used with less frequency over the last years in pediatric cardiac admissions with cardiac arrest. After adjustment for cardiac diagnoses, comorbidities, vasoactive medications, and other resuscitation medications, sodium bicarbonate is independently associated with increased mortality.

## 1. Introduction

Approximately 5000–10,000 children per year in the United States undergo cardiopulmonary resuscitation (CPR) for in-hospital cardiac arrest (IHCA) [1]. The primary diagnosis for IHCA continues to be hypotension and acute respiratory failure [1,2]. The limited evidence of benefit and potential for harm have led the American Heart Association (AHA) to discourage sodium bicarbonate use in recent resuscitation guidelines [3]. Both the 2005 American Heart Association’s (AHA) Advanced Cardiac Life Support (ACLS) and Pediatric Advanced Life Support (PALS) guidelines maintain that routine admiration of sodium bicarbonate has not shown to improve outcome of resuscitation [4]. The 2010 ACLS and PALS guidelines do not recommend use of sodium bicarbonate for cardiac arrest unless the target is hyperkalemia or toxidromes [4].

Although routine use of sodium bicarbonate in pediatric CPR has not been advocated by PALS guidelines, its use during resuscitation remains common. A national database analysis found 68% of pediatric in-hospital cardiac arrests between 2000–2010 received sodium bicarbonate during CPR [4]. The reasoning for its utilization arises from the belief that acidemia impairs myocardial function and diminishes myocardial response to catecholamines [5]. Studies have quoted that pH of less than 7.3 may begin to impair cardiac function, but the majority of these studies are based on shortening fraction of individual sarcomeres isolated in an ex vivo environment. Clinical studies have demonstrated decreased function generally associated with pH less than 7.00. While limited studies have shown improved survival with sodium bicarbonate administration during CPR, overwhelmingly both animal studies [6] and adult studies have shown worsening of cardiac output and failure to improve outcomes of cardiac arrest [2].

The objectives of this study were to characterize the use of sodium bicarbonate in pediatric cardiac admissions in the intensive care unit that experience cardiac arrest, to determine sodium bicarbonate use over the years, and to determine the impact of sodium bicarbonate on length of admissions, billed charges, and inpatient mortality.

## 2. Materials and Methods 

Consent from individual patients was not obtained by the authors of this study, as this study utilized deidentified data from a national database. The study is in compliance with the Helsinki declaration.

### 2.1. Pediatric Health Information System Database

Data for this study were obtained from the Pediatric Health Information System (PHIS). PHIS is an administrative and billing database that contains inpatient, emergency department, ambulatory surgery, and observation data from not-for-profit, tertiary care pediatric hospitals in the United States. The 53 hospitals that contribute data to PHIS are affiliated with the Children’s Hospital Association (Lenexa, KS), a business alliance of children’s hospitals. Data quality and reliability are assured through a joint effort between the Children’s Hospital Association and participating hospitals. For the purposes of external benchmarking, participating hospitals provide discharge/encounter data, including demographics, diagnoses, procedures, and charges. Data are de-identified at the time of data submission, and data are subjected to a number of reliability and validity checks before being included in the database.

### 2.2. Patient Identification

Cardiac admissions in the intensive care unit were identified using international classification of disease-9 (ICD-9) codes for specific cardiac diagnoses that are outlined in Table S1. Cardiac arrest was identified as being present or absent in these admissions using ICD-9 code 427.5. Only admissions with one of the specific cardiac diagnoses and cardiac arrest were deemed appropriate for inclusion. Admissions with and without cardiac surgery were eligible for inclusion, and cardiac surgery was identified using ICD-9 codes outlined in Table S1. We further divided patients that met the inclusion criteria into two subgroups: Patients that received sodium bicarbonate during the admission, and patients that did not.

Any use of the word “admission” in the manuscript from here on out refers to admissions that met these inclusion criteria.

### 2.3. Data Collection

Demographic information such as age and gender was collected for each admission. Admission characteristics such as length of stay, billed charges, and inpatient mortality were also collected. Use of the following vasoactive medications was noted: Epinephrine, norepinephrine, dopamine, dobutamine, milrinone, and vasopressin. Use of calcium chloride or calcium gluconate was also collected. The presence of heart failure, tachyarrhythmia, bradyarrhythmia, pulmonary hypertension, and acute kidney injury was also noted, as these can often complicate pediatric cardiac admissions.

### 2.4. Statistical Analyses

A cross-sectional study was conducted utilizing the Pediatric Health Information System database. Continuous variables are presented as median and range, while categorical variables are presented as absolute frequency and percentage. Continuous variables were analyzed using a Mann–Whitney *U* test, while categorical variables were analyzed using Fisher’s exact test due to the non-normal distribution of the data.

Characteristics of admissions with and without sodium bicarbonate were initially compared by univariate analyses. Next, the frequency of admissions during which sodium bicarbonate was used was compared by year of admission.

Regression analyses were then conducted to determine the impact of sodium bicarbonate use on length of stay, billed charges, and inpatient mortality. Linear regressions were used for length of stay and billed charges, while a logistic regression was used for inpatient mortality. In each of these regressions, one of the three admission characteristics was the dependent variable, while the cardiac diagnoses, comorbidities, and medications listed in Table 1 were entered as independent variables. All variables found in Table 1 were eligible for inclusion and were included in the regression analyses, except for length of stay, cost of stay, and inpatient mortality themselves. Sodium bicarbonate was also included as an independent variable. This was possible due to the large sample size present, and thus, the analyses were adequately powered with a larger number of variables entered into the regression analyses. A total of 48 variables, including use of sodium bicarbonate, were entered as independent variables into the regression models, and with well over 10 events per independent variable, the resulting coefficients were deemed to be well powered as per previously published data regarding statistical methodologies in regards to regression analyses.

It was decided to use regression analyses rather than propensity score matching, as with a higher sample size, regression analyses offer advantages, including the ability to quantify the precise effect of each variable on the endpoint of interest.

All statistical analyses were done utilizing SPSS version 23.0. A *p*-value of less than 0.05 was considered statistically significant. Any use of the word “significant” in this manuscript refers to statistical significance unless otherwise specified.

## 3. Results

### 3.1. Comparison of Admissions with and without Sodium Bicarbonate: Univariate Analyses

A total of 7926 admissions were deemed eligible for inclusion. Of these, 3987 (50.3%) used sodium bicarbonate. There was no significant difference in age or gender between the admissions with and without sodium bicarbonate use. The only significant differences in cardiac lesions were lower frequency of secundum atrial septal defect, systemic venous anomaly, and congenital pulmonary artery anomaly in admissions with sodium bicarbonate used. Cardiac surgery was also significantly less frequent in the admissions with sodium bicarbonate (Table 1).

Trisomy 18, trisomy 21, and 22q11 deletion were significantly less frequent in the sodium bicarbonate admissions, although there were no significant differences in proportion of admissions with trisomy 13 or Turner syndrome. Tachyarrhythmia, bradyarrhythmia, acute kidney injury, and pulmonary hypertension were all significantly less frequent in sodium bicarbonate admissions (Table 1).

All vasoactive medications except dobutamine were used significantly more frequently in sodium bicarbonate admissions. Both calcium gluconate and calcium chloride were also used significantly more frequently in sodium bicarbonate admissions (Table 1).

Length of stay was significantly shorter in sodium bicarbonate admissions (median of 12 days versus 45 days; *p* < 0.01). Billed charges were also significantly lower in sodium bicarbonate admissions (median $240,285 versus $737,917; *p* < 0.01). Inpatient mortality was significantly more frequent in sodium bicarbonate admissions (54.6% versus 35.8%; *p* < 0.01).

### 3.2. Frequency of Sodium Bicarbonate Use by Year of Admission

**Figure 1:**
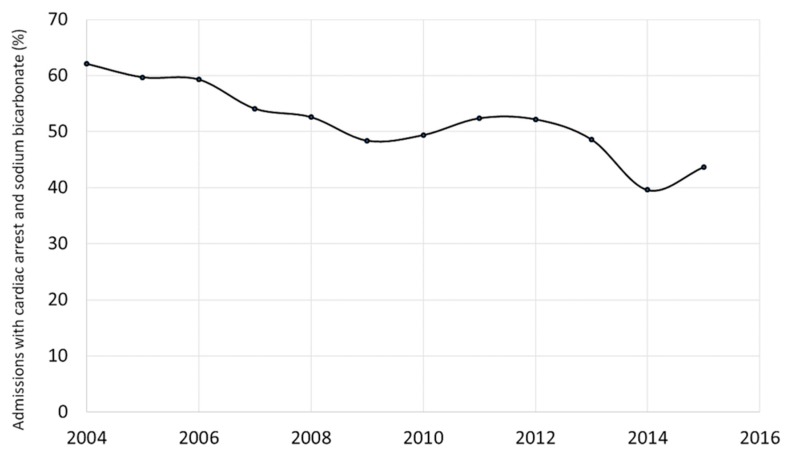
Frequency of sodium bicarbonate use by year of admission.

The use of sodium bicarbonate in pediatric cardiac admissions with cardiac arrest did significantly decline over the study period that spanned from 2004 through the end of 2015. In 2004, 62.1% of admissions utilized sodium bicarbonate compared to 43.7% in 2015 (*p* < 0.01) (Figure 1).

Linear regression analysis with length of stay as the dependent variable and sodium bicarbonate as one of the independent variables demonstrated that sodium bicarbonate was independently associated with a decrease in length of stay (−27.5 days; *p* < 0.01). Linear regression analysis with billed charges as the dependent variable demonstrated that sodium bicarbonate was independently associated with a decrease in billed charges (−$470,906; *p* < 0.01). Logistic regression analysis with inpatient mortality as the independent variable demonstrated that sodium bicarbonate was independently associated with an increase mortality (odds ratio 1.77, 95% confidence interval 1.56–2.01) (Table 2).

As variables other than sodium bicarbonate were included in the regression models, we have provided what other independent variables were significantly associated with increased length of stay, increased billed charges, and increased inpatient mortality. As the aim of this study was to focus on sodium bicarbonate, the findings are not detailed here further in the text, but are available in Table 3.

## 4. Discussion

This study demonstrates that the overall use of sodium bicarbonate from 2004 to 2015 decreased in pediatric cardiac intensive care admissions from 62.1% in 2004 to 43.7% in 2015. Although there has been a decrease trend in its use, it remains a highly used medication in the critical care setting.

Although the use of vasoactive agents has decreased in recent years in children undergoing congenital heart surgery [7], the association between sodium bicarbonate and increased use of all vasoactive medications may not be unexpected, as it may represent patients who developed poor oxygen delivery with lactic acidosis related to hemodynamic instability. It may also reflect the practice that during patient resuscitation situations, clinicians will attempt alternative therapies in an effort to stabilize hemodynamics. Sodium bicarbonate use was recommended in the ACLS guidelines in 1976 and 1980 [8,9], under the assumption that it will counteract the metabolic and respiratory acidosis that cardiac arrest generates due to tissue hypoxia, impaired perfusion, and lactic acid production [10]. However, several recent studies have demonstrated a lack of hemodynamic benefits, among other conflicting findings [5,11].

The finding that sodium bicarbonate therapy during cardiac arrest is associated with increased odds in mortality has been previously demonstrated by other investigators. Lokesh and colleagues performed a randomized controlled trial in a pediatric population utilizing sodium bicarbonate during cardiac arrest. This trial analyzed 55 newborn patients with asphyxia that required resuscitation maneuvers, and did not find benefit in mortality in the sodium bicarbonate group [12]. A more recent study performed by Raymond and colleagues evaluated survival in 3719 pediatric patients that required CPR events. This retrospective study identified a decreased 24-h survival and decrease survival to discharge in the sodium bicarbonate group. Further studies are necessary to determine the cause for the increased association with mortality.

Sodium bicarbonate therapy maybe associated with other adverse effects that may impact patient outcomes. It has been reported that sodium bicarbonate therapy may lead to fluid overload and hyperosmolarity [13]. This finding has been particularly studied in premature neonates in which an association between increased serum osmolarity, elevated arterial carbon dioxide concentrations, and cerebral vascular vasodilation has been found [14]. Sodium bicarbonate therapy may induce hypokalemia and ionized hypocalcemia, which in turn may lead to rhythm disturbances and ventricular dysfunction [15]. Lastly, the 2005 AHA guidelines for CPR warned about the use sodium bicarbonate therapy due to possible reduction in systemic vascular resistance compromising coronary perfusion pressure, extracellular alkalosis inhibiting oxygen release from tissue by shifting oxyhemoglobin saturation curve, and increasing carbon dioxide production that can freely enter cells and may contribute to intracellular acidosis [16]. In fact, the 2015 AHA PALS guidelines discouraged the routine administration of sodium bicarbonate in cardiac arrest in children [17], and concluded that the risks of using sodium bicarbonate outweigh the benefits of its buffer effect, and its therapeutic use is limited to specific situations, such as: Ventricular arrhythmias (1–2 mEq/kg, in addition to standard treatment), tricyclic antidepressants intoxication (1–2 mEq/kg intravenous (IV) boluses until pH is over 7.45), or during hyperkalemic cardiac arrest [18]. In 2018, the scientific statement from the AHA Congenital Cardiac Defects Committee revised the CPR guidelines specifically for infants and children with cardiac disease [19]. The guidelines extended the indications of sodium bicarbonate therapy for patients with a pacemaker with severe metabolic acidosis to avoid a lack of pacemaker capture, and in patients with severe metabolic acidosis associated with myocardial dysfunction. These changes in practice could have impacted the decrease of patients who receive this therapy, and that is demonstrated in Figure 1; furthermore, it could have also affected the characteristics of the patients to critical ones.

While this study benefits from a large number of admissions, there are limitations to this analysis. There are data not collected in the database which could help understand the temporal relationship between interventions. As a result, all the findings here are associations and not causative. The timing of sodium bicarbonate administration is not recorded, so one cannot tell at which point the sodium bicarbonate was administered precisely in relation to the arrest. It also appears that the sodium bicarbonate group may represent a sicker cohort of patients. While this is true, regression analyses factored in multiple characteristics that should have helped to account for this, including—but not limited to—the use of vasoactive medications. This study is also constrained by limitations inherent to administrative databases such as miscoding or undercoding. Inaccurate or incomplete coding may lead to misreporting, may affect outcome analyses, and are difficult to account for by statistical methodology. Data quality and reliability are assured through a joint effort between the Children’s Hospital Association and participating hospitals, and data are subjected to a number of reliability and validity checks. The multi-institutional data utilized for this study represent the perspective on outcomes only in participating centers and may not be representative of or generalizable to non-participating centers and non-US centers. Outcomes were only measured for the index hospitalization; data from subsequent hospitalizations were not evaluated. Another limitation is that the shorter length of stay and cost of hospitalization might be due to an early mortality rather than the effects of sodium bicarbonate. Finally, these analyses are limited to direct hospital costs and do not include physician fees and other costs carried by patients and families.

Nonetheless, this study does present data that characterize the use of sodium bicarbonate. The increased inpatient mortality is provocative and is similar to findings from previous studies. Prudent use of sodium bicarbonate is warranted. Decreasing frequency of its use does appear to demonstrate that this viewpoint is being increasingly accepted in clinical practice.

## 5. Conclusions

Sodium bicarbonate is being used with less frequency in recent years in pediatric cardiac admissions with cardiac arrest. After adjustment for cardiac diagnoses, comorbidities, vasoactive medications, and other resuscitation medications, sodium bicarbonate is independently associated with increased mortality.

## Figures and Tables

**Table 1:** Comparison of admissions with and without sodium bicarbonate: Univariate analyses.

	No Sodium Bicarbonate (*n* = 3939)	Sodium Bicarbonate (*n* = 3987)	Odds Ratio (95% Confidence Interval)	*p*-Value
Gender (male)	2167 (55.0)	2190 (54.9)	-	0.99
Age (years)	0 (0–17)	0 (0–17)	-	0.28
Cardiac lesion				
Primum atrial septal defect	26 (0.7)	17 (0.4)	0.64 (0.34–1.19)	0.15
Secundum atrial septal defect	1170 (29.7)	1080 (27.1)	0.87 (0.79–0.97)	0.01
Ventricular septal defect	555 (14.1)	549 (13.8)	0.97 (0.85–1.10)	0.68
Double outlet right ventricle	239 (6.1)	236 (5.9)	0.97 (0.80–1.17)	0.78
Tetralogy of Fallot	190 (4.8)	181 (4.5)	0.93 (0.76–1.15)	0.55
Pulmonary atresia	119 (3.0)	106 (2.7)	0.87 (0.67–1.14)	0.33
Atrioventricular septal defect	297 (7.5)	3306 (7.7)	1.01 (0.86–1.20)	0.82
Transposition	124 (3.1)	144 (3.6)	1.15 (0.90–1.47)	0.25
Congenitally corrected transposition	26 (0.7)	36 (0.9)	1.37 (0.82–2.27)	0.22
Common arterial trunk	59 (1.5)	54 (1.4)	0.90 (0.62–1.31)	0.59
Ebstein anomaly	50 (1.3)	51 (1.3)	1.01 (0.68–1.49)	0.96
Hypoplastic left heart syndrome	513 (13.0)	483 (12.1)	0.92 (0.80–1.05)	0.22
Functionally univentricular heart other than HLHS	183 (4.6)	150 (3.8)	0.80 (0.64–1.00)	0.05
Coarctation of the aorta	264 (6.7)	252 (6.3)	0.93 (0.78–1.12)	0.49
Interrupted aortic arch	43 (1.1)	39 (1.0)	0.89 (0.57–1.38)	0.61
Partial anomalous pulmonary venous connection	39 (1.0)	40 (1.0)	1.01 (0.65–1.57)	0.95
Total anomalous pulmonary venous connection	100 (2.5)	106 (2.7)	1.04 (0.79–1.38)	0.73
Systemic venous anomaly	158 (4.0)	113 (2.8)	0.69 (0.54–0.89)	<0.01
Congenital tricuspid stenosis	83 (2.1)	83 (2.1)	0.98 (0.72–1.34)	0.93
Congenital mitral stenosis	57 (1.4)	49 (1.2)	0.84 (0.57–1.24)	0.39
Congenital pulmonary stenosis	99 (2.5)	115 (2.9)	1.15 (0.87–1.51)	0.30
Congenital aortic stenosis	46 (1.2)	65 (1.6)	1.40 (0.95–2.05)	0.08
Congenital subaortic stenosis	52 (1.3)	46 (1.2)	0.87 (0.58–1.30)	0.50
Congenital pulmonary artery anomaly	184 (4.7)	110 (2.8)	0.57 (0.45–0.73)	<0.01
Congenital coronary anomaly	130 (3.3)	134 (3.4)	1.01 (0.79–1.30)	0.88
Cardiac surgery	1456 (37.0)	1135 (28.5)	0.67 (0.61–0.74)	<0.01
Trisomy 13	13 (0.3)	9 (0.2)	0.68 (0.29–1.60)	0.37
Trisomy 18	31 (0.8)	17 (0.4)	0.54 (0.29–0.97)	0.03
Trisomy 21	180 (4.6)	218 (5.5)	1.20 (0.98–1.47)	0.06
Turner syndrome	11 (0.3)	11 (0.3)	0.98 (0.42–2.28)	0.97
22q11 deletion	94 (2.4)	61 (1.5)	0.63 (0.45–0.88)	<0.01
Isomerism	158 (4.0)	111 (2.8)	0.68 (0.53–0.87)	<0.01
Heart failure	285 (7.2)	283 (7.1)	0.98 (0.82–1.16)	0.81
Tachyarrhythmia	1193 (30.3)	1047 (26.3)	0.82 (0.74–0.90)	<0.01
Bradyarrhythmia	380 (9.6)	323 (8.1)	0.82 (0.70–0.96)	0.01
Acute kidney injury	1428 (36.3)	1319 (33.1)	0.86 (0.79–0.95)	<0.01
Pulmonary hypertension	700 (17.8)	478 (12.0)	0.63 (0.55–0.71)	<0.01
Epinephrine	2491 (63.2)	3661 (91.8)	6.52 (6.72–7.42)	<0.01
Norepinephrine	569 (14.4)	712 (17.9)	1.28 (1.14–1.45)	<0.01
Dopamine	1831 (46.5)	2540 (63.7)	2.02 (1.84–2.21)	<0.01
Dobutamine	430 (10.9)	475 (11.9)	1.10 (0.96–1.26)	0.16
Milrinone	2125 (53.9)	2372 (59.5)	1.25 (1.14–1.37)	<0.01
Vasopressin	93 (2.4)	830 (20.8)	10.87 (8.73–13.54)	<0.01
Calcium gluconate	568 (14.4)	1884 (47.3)	5.31 (4.77–5.92)	<0.01
Calcium chloride	691 (17.5)	2830 (71.0)	11.49 (10.33–12.79)	<0.01
Length of stay	45 (1–915)	12 (1–262)	-	<0.01
Cost of stay	737,917	240,285	-	<0.01
Mechanical ventilation	3689 (93.7)	3824 (95.9)	1.59 (1.29–1.94)	<0.01
Extracorporeal membrane oxygenation	1116 (28.3)	973 (24.4)	0.81 (0.73–0.90)	<0.01
Inpatient mortality	1411 (35.8)	2177 (54.6)	2.15 (1.96–2.35)	<0.01

**Table 2:** Effect of sodium bicarbonate on length of stay, billed charges, and inpatient mortality: Regression analyses.

	Length of Stay (days)	Billed Charges (US Dollars)	Inpatient Mortality (Odds Ratio and 95% Confidence Interval)
Sodium bicarbonate	−27.5 (*p* < 0.01)	−470,906 (*p* < 0.01)	1.77 (1.56–2.01)

**Table 3:** Independent variables other than sodium bicarbonate significantly associated with increased length of stay, billed charges, and inpatient mortality.

Length of Stay (Days)	Billed Charges (USD)	Inpatient Mortality
Younger age Higher RACHS score Secundum atrial septal defect Tetralogy of Fallot Hypoplastic left heart syndrome Coarctation of the aorta Systemic venous anomaly Isomerism Acute kidney injury Pulmonary hypertension Hypothyroidism ECMO Dobutamine Milrinone	Higher RACHS score Secundum atrial septal defect Tetralogy of Fallot Hypoplastic left heart syndrome Coarctation of the aorta Systemic venous anomaly Tachyarrhythmia Bradyarrhythmia Acute kidney injury Pulmonary hypertension Hypothyroidism ECMO Dopamine Milrinone	Younger age Higher RACHS score Atrioventricular septal defect Hypoplastic left heart syndrome Total anomalous pulmonary venous return Systemic venous anomaly Congenital coronary anomaly Isomerism Acute kidney injury Pulmonary hypertension Syndrome Norepinephrine Dopamine Dobutamine Vasopressin Calcium chloride ECMO

RACHS: risk adjustment in congenital heart surgery; ECMO: extracorporeal membrane oxygenation.

## Supplementary Materials

Supplementary materials can be accessed at https://www.mdpi.com/2227-9067/6/12/136/s1.
