# Supplementary material for: Use of Sodium Bicarbonate During Pediatric Cardiac Admissions with Cardiac Arrest: Who Gets It and What Does It Do?
Source: Children (Basel). 2019 Dec 16;6(12):136. doi: 10.3390/children6120136 (PMC6955993; doi:10.3390/children6120136)
Supplement: Supplementary file 1 [file children-06-00136-s001.pdf]

Table S1. International Classification of Disease-9 Description for Congenital Heart malformations.

ICD9	ICD9 Description CHD	ICD9	ICD9 Description Surgical Procedures
426.0	Atrioventricular block complete	35.00	Closed heart valvotomy, unspecified valve
426.1	Atrioventricular block, other and unspecified	35.01	Closed heart valvotomy, aortic valve
426.10	Atrioventricular block NOS	35.02	Closed heart valvotomy, mitral valve
426.11	Atrioventricular block-1st degree	35.03	Closed heart valvotomy, pulmonary valve
426.12	Atrioventricular block-mobitz ii	35.04	Closed heart valvotomy, tricuspid valve
426.13	Atrioventricular block-2nd degree NEC	35.10	Open heart valvuloplasty without replacement, unspecified valve
426.50	Bundle branch block NOS	35.11	Open heart valvuloplasty of aortic valve without replacement
427.0	Paroxysmal atrial tachycardia	35.12	Open heart valvuloplasty of mitral valve without replacement
427.1	Paroxysmal ventricular tachycardia	35.13	Open heart valvuloplasty of pulmonary valve without replacement
427.2	Paroxysmal tachycardia NOS	35.14	Open heart valvuloplasty of tricuspid valve without replacement
427.3	Atrial fibrillation and flutter	35.20	Replacement of unspecified heart valve
427.31	Atrial fibrillation	35.21	Replacement of aortic valve with tissue graft
427.32	Atrial flutter	35.22	Other replacement of aortic valve
427.4	Ventricular fibrillation and flutter	35.23	Replacement of mitral valve with tissue graft
427.41	Ventricular fibrillation	35.24	Other replacement of mitral valve
427.42	Ventricular flutter	35.25	Replacement of pulmonary valve with tissue graft
428.0	Congestive heart failure NOS	35.26	Other replacement of pulmonary valve
428.1	Left heart failure	35.27	Replacement of tricuspid valve with tissue graft
428.2	Systolic heart failure	35.28	Other replacement of tricuspid valve
428.20	Systolic heart failure NOS	35.31	Operations on papillary muscle
428.21	Acute systolic heart failure	35.32	Operations on chordae tendineae
428.22	Chronic systolic heart failure	35.33	Annuloplasty
428.23	Acute on chronic systolic heart failure	35.34	Infundibulectomy
428.3	Diastolic heart failure	35.35	Operations on trabeculae carnae cordis
428.30	Diastolic heart failure NOS	35.39	Operations on other structures adjacent to valves of heart
428.31	Acute diastolic heart failure	35.41	Enlargement of existing atrial septal defect
428.32	Chronic diastolic heart failure	35.42	Creation of septal defect in heart
428.33	Acute on chronic diastolic heart failure	35.50	Repair of unspecified septal defect of heart with prosthesis
428.4	Combined systolic and diastolic heart failure	35.51	Repair of atrial septal defect with prosthesis, open technique
428.40	Systolic/diastolic heart failure NOS	35.52	Repair of atrial septal defect with prosthesis, closed technique
428.41	Acute systolic/diastolic heart failure	35.53	Repair of ventricular septal defect with prosthesis
428.42	Chronic systolic/diastolic heart failure	35.54	Repair of endocardial cushion defect with prosthesis
428.43	Acute/chronic systolic/diastolic heart failure	35.60	Repair of unspecified septal defect of heart with tissue graft
428.9	Heart failure NOS	35.61	Repair of atrial septal defect with tissue graft
584.9	Acute kidney failure NOS	35.62	Repair of ventricular septal defect with tissue graft
585.9	Chronic kidney disease NOS	35.63	Repair of endocardial cushion defect with tissue graft
745.0	Common arterial trunk	35.70	Other and unspecified repair of unspecified septal defect of heart
745.10	Complete transposition of the great vessels	35.71	Other and unspecified repair of atrial septal defect
745.11	Double outlet right ventricle	35.72	Other and unspecified repair of ventricular septal defect

745.12	Congenitally corrected transposition of the great vessels	35.73	Other and unspecified repair of endocardial cushion defect
745.19	Transposition of the great vessels NEC	35.81	Total repair of tetralogy of Fallot
745.5	Secundum atrial septal defect	35.82	Total repair of total anomalous pulmonary venous connection
745.60	Endocardial cushion defect NOS	35.83	Total repair of truncus arteriosus
745.61	Ostium primum defect	35.84	Total correction of transposition of great vessels, not elsewhere classified
745.69	Atrioventricular septal defect	35.91	Interatrial transposition of venous return
746.01	Congenital pulmonary valve atresia	35.92	Creation of conduit between right ventricle and pulmonary artery
746.1	Congenital tricuspid atresia/stenosis	35.93	Creation of conduit between left ventricle and aorta
746.2	Ebstein's anomaly	35.94	Creation of conduit between atrium and pulmonary artery
746.7	Hypoplastic left heart syndrome	35.95	Revision of corrective procedure on heart
746.85	Coronary artery anomaly	35.98	Other operations on septa of heart
747.41	Total anomalous pulmonary venous connection	35.99	Other operations on valves of heart
747.42	Partial anomalous pulmonary venous connection	36.99	Other operations on vessel of heart
		37.33	Excision or destruction of other lesion or tissue of heart
		37.5	Heart replacement procedures
		37.51	Heart transplantation
		37.52	Implantation of total replacement heart system
		39.0	Systemic to pulmonary artery shunt
		39.21	Caval-pulmonary artery anastomosis

Note: CHD, Congenital Heart Disease; ICD9, International Classification of Disease-9; NEC, not elsewhere classifiable; NOS, not otherwise specified.
